# Development and validation of a Comprehensive Hematological Scoring System for predicting overall survival in patients with soft tissue sarcomas: a comparison with NLR and PLR

**DOI:** 10.3389/fonc.2025.1505485

**Published:** 2025-08-01

**Authors:** Ying Qiu, Jiquan Liu, Shuang Chai, Lili Liu, Longqing Li, Yan Zhang

**Affiliations:** ^1^ Department of Orthopedics, Henan Luoyang Orthopedic Hospital (Henan Provincial Orthopedic Hospital), Zhengzhou, Henan, China; ^2^ Department of Orthopedics, The First Affiliated Hospital of Zhengzhou University, Zhengzhou University, Zhengzhou, Henan, China

**Keywords:** soft tissue sarcoma (STS), Comprehensive Hematological Scoring System (CHSS), neutrophil-to-lymphocyte ratio (NLR), platelet-to-lymphocyte ratio (PLR), prognosis

## Abstract

**Background:**

Soft tissue sarcomas (STS) are rare malignancies with high relapse/metastasis risks and limited treatment efficacy. Current biomarkers like neutrophil-to-lymphocyte ratio (NLR) and platelet-to-lymphocyte ratio (PLR) lack comprehensive prognostic value due to their reliance on limited hematological parameters.

**Methods:**

This retrospective study analyzed 206 STS patients (2016–2023) to develop a Composite Hematological Scoring System (CHSS) integrating 19 pretreatment markers. LASSO regression selected key variables (glucose, CRP, LDL-C, HDL-C, albumin, platelets, hemoglobin, lymphocytes), weighted by coefficients. CHSS’s prognostic performance was compared to NLR/PLR via Kaplan-Meier, time-dependent ROC, and Cox regression analyses. A nomogram combining CHSS with clinical variables was validated using C-index, calibration, and decision curves.

**Results:**

CHSS outperformed NLR/PLR in predicting overall survival (OS) across all timepoints. High CHSS patients had significantly worse OS (HR=6.197, P<0.001). Multivariate analysis confirmed CHSS, age, tumor size, and FNCLCC grade as independent predictors. The CHSS-based nomogram achieved a C-index of 0.79, with accurate 3-/5-year OS calibration.

**Conclusion:**

CHSS integrates inflammation, metabolism, and nutrition markers to provide superior prognostic stratification for STS patients compared to NLR/PLR. Its integration into a nomogram supports personalized management, though multicenter validation is needed.

## 1 Introduction

Soft tissue sarcomas (STS) are rare solid cancers originating from mesenchymal tissues —including muscle, adipose, bone, and fibrous tissues—comprising approximately 1% of adult malignancies and exhibiting an annual incidence of 4–5 per 100,000 individuals (1). Liposarcoma (LPS), leiomyosarcoma (LMS), and undifferentiated pleomorphic sarcoma (UPS) represent the most prevalent subtypes, although the WHO classification system recognizes over 70 distinct histopathologic subtypes (2–4). A critical clinical challenge lies in the high rates of local recurrence and distant metastasis, occurring in 25–50% of patients, with risk stratification dependent on tumor stage and histologic subtype (5). For locally advanced and metastatic STS, first-line chemotherapies such as doxorubicin and ifosfamide remain standard-of-care, yet demonstrate limited efficacy, yielding a median overall survival (OS) of merely 10–15 months in metastatic cases (6, 7). Over the past decade, therapeutic paradigms have evolved from uniform protocols to histology-driven algorithms, incorporating tumor subtype- and stage-adjusted surgical and multimodal interventions (8).

Advances in tumor biology have established that systemic inflammation, metabolic dysregulation, and nutritional status are intrinsically linked to tumor aggressiveness and clinical outcomes (9–11). This understanding has catalyzed the emergence of prognostic biomarkers, including the neutrophil-to-lymphocyte ratio (NLR), platelet-to-lymphocyte ratio (PLR), prognostic nutritional index (PNI), and Controlling Nutritional Status Score (CONUT) (12–15). While these hematologic indices reflect inflammatory or nutritional derangements and enable partial prediction of oncologic prognosis and therapeutic responses, most rely on limited parameter combinations—for instance, NLR is derived solely from absolute neutrophil and lymphocyte counts—thereby limiting their ability to fully exploit hematologic data (16). It is reasonable to speculate that a multidimensional scoring system integrating comprehensive laboratory parameters may offer superior prognostic predictive capacity.

In this study, we retrospectively analyzed 19 pretreatment hematologic parameters spanning inflammation, nutrition, metabolism, and coagulation, constructing a composite prognostic score via Least Absolute Shrinkage and Selection Operator (LASSO) COX proportional hazards regression analysis. This novel scoring system was benchmarked against two conventional hematologic markers (NLR and PLR) to evaluate the comparative prognostic utility of multidimensional versus simplified biomarker approaches.

## 2 Patients and methods

### 2.1 Patients

This retrospective cohort study analyzed the clinical data of patients with STS treated at the Musculoskeletal Tumor Center of Zhengzhou University First Affiliated Hospital from June 2016 to June 2023. The inclusion criteria for patients were as follows: 1. Histopathologically confirmed STS; 2. Complete pretreatment hematologic profiles; 3. receipt of institutionally approved standard therapies. The exclusion criteria: 1. Postsurgical recurrence; 2. Concurrent hematologic disorders; 3. Secondary malignancies. All enrolled patients underwent surgical treatment and were followed up regularly until death or June 2023. The ethics committee of Zhengzhou University First Affiliated Hospital approved this study, and each participant signed a written informed consent form.

### 2.2 Data collection and analysis

Nineteen pretreatment laboratory parameters were collected: neutrophils, lymphocytes, monocytes, red blood cells, red cell distribution width, platelets, hemoglobin, albumin, globulin, glucose, triglycerides, high-density lipoprotein (HDL), low-density lipoprotein (LDL), activated partial thromboplastin time (APTT), prothrombin time (PT), fibrinogen, thrombin time (TT), and C-reactive protein (CRP). The optimal cutoff values for each indicator were calculated using receiver operating characteristic (ROC) curves, converting all indicators into binary variables. Clinical variables including age, gender, body mass index (BMI), tumor size, tumor location, and the French Federation Nationale des Centres de Lutte Contre le Cancer sarcoma grade (FNCLCC) were extracted from electronic medical records. Overall survival (OS) was defined as the interval from diagnosis to death or last follow-up.

### 2.3 Comparison of prognostic value of NLR/PLR/CHSS in STS patients

NLR = Neutrophil/Lymphocyte; PLR = Platelet/Lymphocyte. The construction method of the CHSS score is as follows: first, variables with prognostic value in STS patients were screened using logistic regression (P<0.05). Subsequently, LASSO regression analysis was performed to reduce dimensionality of the selected variables and assign a coefficient to each variable. The CHSS score is the sum of all variables multiplied by their respective coefficients. The optimal cutoff value for the CHSS score was calculated using ROC analysis. Kaplan-Meier survival curves were plotted to evaluate the prognostic value of the three biomarkers in predicting overall survival in STS patients. The predictive capabilities of the three biomarkers were compared using time-dependent ROC curves. Subgroup analyses were conducted to compare the stability of the predictive abilities of the three biomarkers.

### 2.4 Construction and evaluation of the CHSS-based nomogram for STS

CHSS was integrated with clinical covariates to identify independent OS predictors via multivariable Cox regression. A prognostic nomogram was constructed using significant predictors, with discriminative performance evaluated by Harrell’s concordance index (C-index) and calibration curves. Decision curve analysis (DCA) and clinical impact curves quantified clinical net benefit.

### 2.5 Statistical analysis

The Kolmogorov-Smirnov test was used to determine whether continuous variables follow a normal distribution. Based on the normality, either the t-test or the Mann-Whitney U test was used to assess differences between continuous variables. The chi-squared test or Fisher’s exact test was used to evaluate differences in categorical variables, depending on the sample size in each group. All statistical analyses were performed using R software version 4.4.0 (Vienna Institute of Statistics and Mathematics, Austria). A P value of < 0.05 was considered statistically significant.

## 3 Results

### 3.1 Patient characteristics

The cohort comprised 206 STS patients (108 males, 98 females) with a mean age of 49.7 ± 13.2 years (range: 26–77). FNCLCC grading classified 145 patients (70.4%) as grade 3 and 61 (29.6%) as grade 2. Tumor distribution included upper limbs (n=31, 15.0%), lower limbs (n=138, 67.0%), and trunk (n=37, 18.0%). Tumor size stratification revealed 27 patients (13.1%) with lesions <5 cm, 99 (48.1%) with 5–10 cm tumors, and 80 (38.8%) with tumors >10 cm. At final follow-up (June 2023), 44 deaths (21.4%) were recorded (**Table 1**).

**Table 1:** Patients demographics.

Variable	CHSS Low Risk (N = 141)	CHSS High Risk (N = 65)	P-value
Overall survival			
Mean (SD)	1600 (734)	1180 (812)	< 0.001
Gender			
Female	66 (46.8%)	32 (49.2%)	0.862
Male	75 (53.2%)	33 (50.8%)	
Age			
Mean (SD)	49.4 (13.7)	50.2 (12.3)	0.665
FNCLCC			
Stage 2	33 (23.4%)	8 (12.3%)	0.0957
Stage 3	108 (76.6%)	57 (87.7%)	
TumorLocation			
Upper extremity	22 (15.6%)	9 (13.8%)	0.429
Lower extremity	97 (68.8%)	41 (63.1%)	
Trunk	22 (15.6%)	15 (23.1%)	
TumorSize			
T<5 cm	17 (12.1%)	10 (15.4%)	0.593
5 cm<T<10 cm	71 (50.4%)	28 (43.1%)	
T>10cm	53 (37.6%)	27 (41.5%)	
BMI			
Abnormal	45 (31.9%)	22 (33.8%)	0.908
Normal	96 (68.1%)	43 (66.2%)	

### 3.2 Comparison of prognostic value of NLR/PLR/CHSS in STS patients

**Figure 1:**
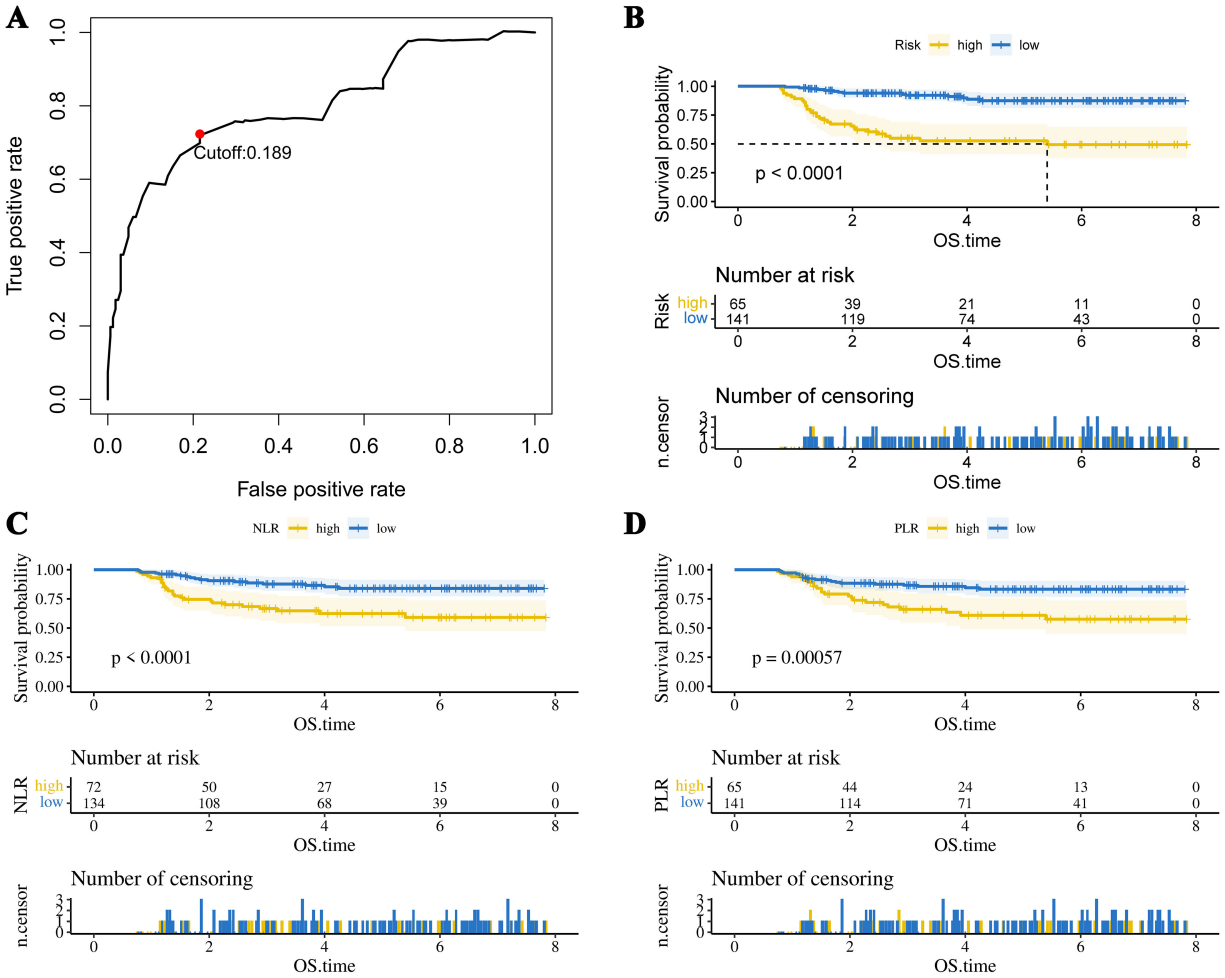
**(A)** The optimal cutoff value of the CHSS score. The KM survival curves show overall survival in patients grouped by **(B)** CHSS, **(C)** NLR, and **(D)** PLR.

**Figure 2:**
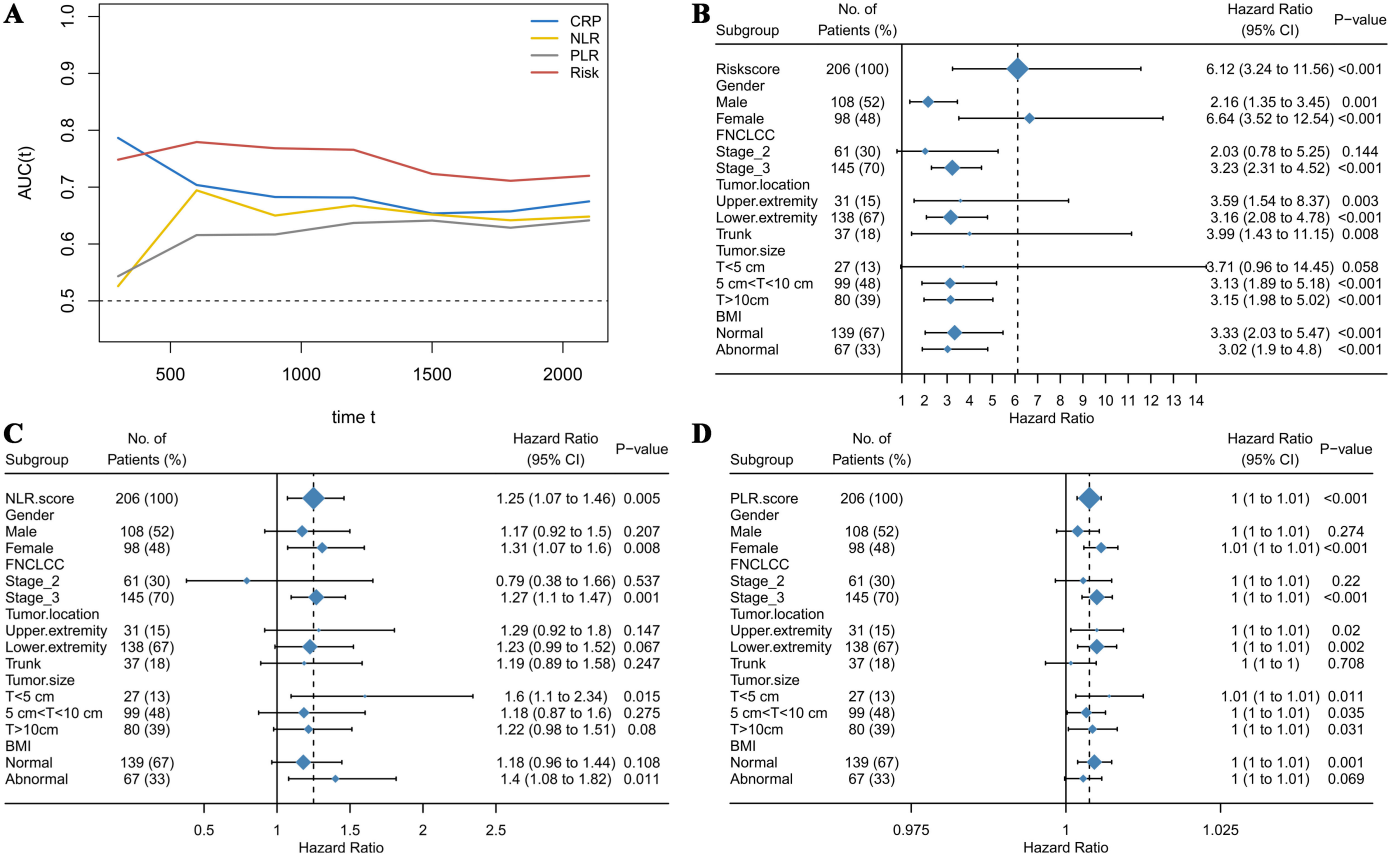
**(A)** The time-dependent ROC curves demonstrate the predictive abilities of different biomarkers; **(B)** The forest plot illustrates the predictive ability of CHSS across different subgroups; **(C)** The forest plot illustrates the predictive ability of NLR across different subgroups; **(D)** The forest plot illustrates the predictive ability of PLR across different subgroups.

The optimal cutoff values for 19 test results in STS patients are shown in **Table 2**. Eight parameters—glucose, CRP, LDL-C, HDL-C, albumin, platelet count (PLT), hemoglobin (HB), and lymphocyte count—were significantly associated with prognosis and incorporated into CHSS. **Table 3** presents the coefficients of the aforementioned test results in the CHSS score. The CHSS cutoff (0.189) stratified patients into high- versus low-risk groups (**Figure 1A**), with significantly worse OS in high-CHSS patients (log-rank P < 0.001). Similarly, patients in the high NLR group and high PLR group had lower overall survival than their respective controls (P < 0.001) (**Figures 1B–D**). The time-dependent ROC curve results indicated that the predictive ability of CHSS was superior to that of NLR/PLR at all time points, and in most instances, it outperformed the constituent indicator CRP (**Figure 2A**). Subgroup analysis results showed that CHSS demonstrated significant predictive ability in the majority of subgroups, whereas NLR/PLR showed limited generalizability (**Figures 2B–D**).

**Table 2:** Univariate logistic results under best roc cutoff.

Variable	Auc	Cutoff	Logistic.Pvalue	Logistic.OR
NLR	0.605	2.851	<0.001	3.643 (1.825-7.271)
PLR	0.633	148.792	0.001	3.129 (1.573-6.227)
LMR	0.538	4.448	0.121	0.560 (0.269-1.166)
PLT	0.601	213	0.019	2.252 (1.145-4.431)
Neutrophil	0.525	4.01	0.183	1.577 (0.807-3.082)
Lymphocyte	0.589	2.08	0.032	0.364 (0.145-0.917)
Monocytes	0.516	0.44	0.265	0.684 (0.350-1.335)
RBC	0.543	3.98	0.082	0.551 (0.282-1.079)
HB	0.527	95	0.022	0.339 (0.134-0.857)
Albumin	0.578	41.3	0.018	0.428 (0.211-0.865)
RDW	0.548	13.7	0.086	1.840 (0.918-3.688)
Globulin	0.584	23.6	0.058	2.029 (0.975-4.222)
Glucose	0.568	5.93	0.001	3.733 (1.675-8.322)
Triglycerides	0.528	1.78	0.057	0.238 (0.054-1.043)
Cholesterol	0.555	4.71	0.052	0.134 (0.018-1.017)
HDL-C	0.573	1.35	0.014	0.316 (0.126-0.793)
LDL-C	0.612	1.97	0.009	2.667 (1.283-5.542)
APTT	0.554	30.4	0.081	1.826 (0.929-3.589)
PT	0.565	11.3	0.09	1.861 (0.907-3.815)
TT	0.515	17.2	0.154	1.839 (0.796-4.251)
FIB	0.528	4.04	0.114	1.845 (0.864-3.943)
CRP	0.629	9.1	0.004	3.080 (1.427-6.649)

**Table 3:** Univariate logistic results under best roc cutoff.

Variable	Coef
PLT	0.567091
Lymphocyte	-0.47881
HB	-0.5132
Albumin	-0.78245
Glucose	0.708249
HDL-C	-0.60477
LDL-C	0.498902
CRP	0.971148

### 3.3 Univariate analysis and multivariate analysis

**Figure 3:**
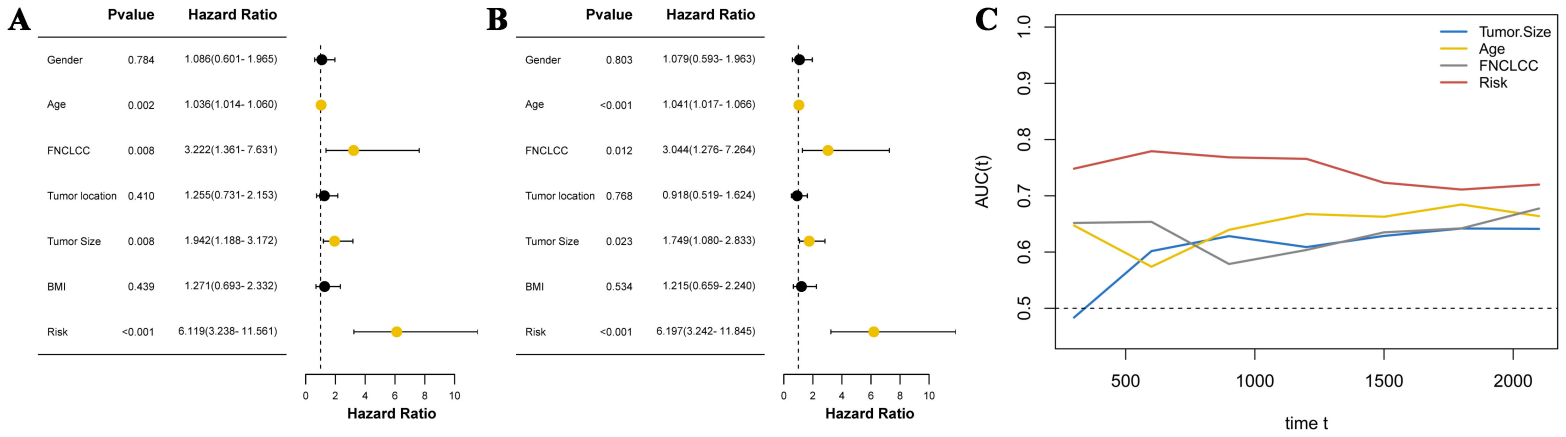
**(A)** The forest plot shows the univariate analysis results of CHSS and clinical variables; **(B)** The forest plot shows the multivariate analysis results of CHSS and clinical variables; **(C)** The time-dependent ROC curve demonstrates the predictive ability of independent prognostic factors.

Cox regression analysis was used to explore independent prognostic factors in STS patients. Univariate analysis revealed that age (hazard ratio (HR) = 1.036 (95% confidence interval (CI) 1.014–1.060), P = 0.002), FNCLCC (HR = 3.222 (1.361–7.631), P = 0.008), tumor size (HR = 1.942 (1.188–3.172), P = 0.008), and CHSS (HR = 6.119 (3.238–11.561), P < 0.001) were associated with OS in STS patients (**Figure 3A**). The results of the multivariate analysis indicated that age (HR = 1.041 (1.017–1.066), P < 0.001), FNCLCC (HR = 3.044 (1.276–7.264), P = 0.012), tumor size (HR = 1.749 (1.080–2.833), P = 0.023), and CHSS (HR = 6.197 (3.242–11.845), P < 0.001) were all independent prognostic factors for STS patients (**Figure 3B**). CHSS consistently outperformed other variables in time-dependent ROC comparisons (**Figure 3C**).

### 3.4 Construction and validation of CHSS-based nomogram

**Figure 4:**
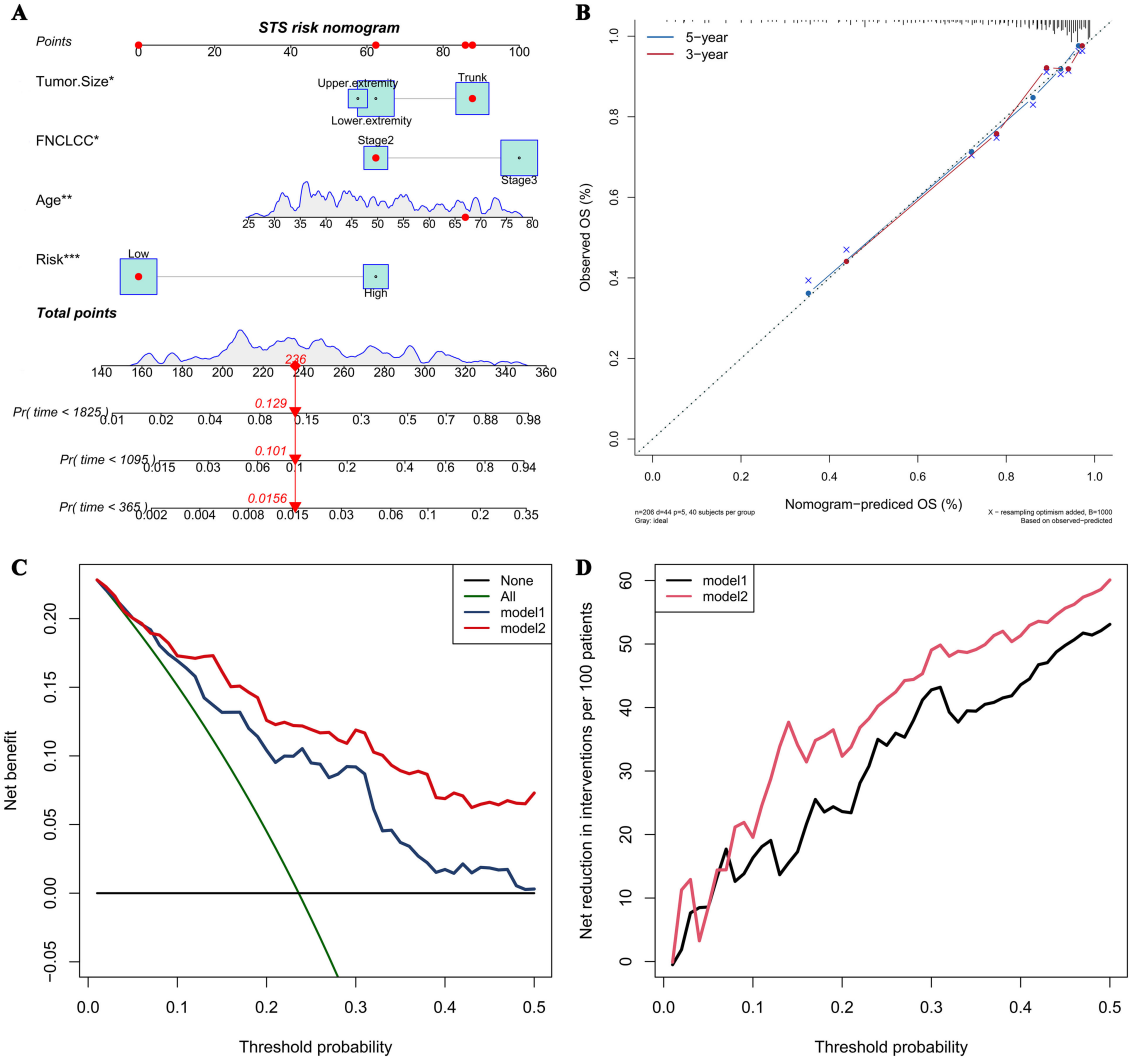
**(A)** The nomogram predicting overall survival in soft tissue sarcoma patients based on independent risk factors; **(B)** The calibration curve of the nomogram; **(C)** The net benefit curve of the nomogram; **(D)** The net reduction curve of the nomogram.

A prognostic nomogram integrating four independent predictors (CHSS, age, FNCLCC grade, tumor size) was developed (**Figure 4A**). The model demonstrated excellent discrimination (C-index=0.79) and calibration accuracy for 3-/5-year OS predictions (**Figure 4B**). Decision curve analysis revealed greater net clinical benefit for Model 2 (CHSS + clinical variables) versus Model 1 (clinical variables alone) across threshold probabilities (**Figures 4C, D**).

## 4 Discussion

This single-center retrospective study evaluated two biomarker development paradigms in 206 STS patients. While both conventional biomarkers (NLR/PLR) and the CHSS demonstrated prognostic utility, time-dependent ROC analyses revealed CHSS’s superior predictive accuracy and stability across follow-up intervals. CHSS was identified as an independent prognostic factor for STS patients, and the nomogram based on CHSS could reliably predict 3-year and 5-year overall survival in STS patients.

In the past decade, accumulating evidence has validated the prognostic value of NLR and PLR in various cancers, including lung cancer, breast cancer, thyroid cancer, and liver cancer (17–22). Furthermore, these inflammatory makers demonstrate prognostic relevance in STS subtypes, particularly liposarcoma (23–28). Emerging evidence further highlights their potential in predicting therapeutic responses, as exemplified by trabectedin outcomes in STS patients (29). NLR’s role as a marker for predicting cancer patient survival may be attributed to its reflection of the balance between the body’s inflammatory and immune status (30). Neutrophils are key effector cells in the inflammatory response and are often elevated in cancer patients, indicating a significant inflammatory reaction. Neutrophils promote tumor progression by releasing cytokines and growth factors, which enhance tumor angiogenesis, suppress immune surveillance, and promote the growth and invasiveness of tumor cells (31, 32). On the other hand, lymphocytes are crucial cells in the immune system responsible for anti-tumor responses (33). A decrease in lymphocytes usually reflects a state of immunosuppression, indicating impaired immune surveillance (34, 35). This immunosuppressive state hinders the body’s ability to effectively eliminate tumor cells, increasing the risk of tumor recurrence and metastasis. A high NLR reflects a combination of increased neutrophils and decreased lymphocytes, indicating a state of both heightened inflammation and weakened anti-tumor immunity. Additionally, experimental tumor therapies that reduce neutrophil counts have further solidified the association between elevated neutrophils and poor prognosis in cancer patients (36, 37).

Similar to NLR, a high PLR also reflects an imbalance between tumor-promoting factors and anti-tumor immune responses (23, 38). For example, platelets can release pro-angiogenic factors such as vascular endothelial growth factor to promote tumor growth (39, 40). However, these biomarkers only incorporate a very limited portion of the hematological test results, making it difficult to fully capture the true value of these tests. In our study, we collected up to 19 blood test results, including neutrophil count, lymphocyte count, monocyte count, and C-reactive protein, which are related to inflammation and immunity; glucose, albumin, triglycerides, HDL-C, LDL-C, and other indicators associated with nutritional status and metabolism; as well as APTT, PT, TT, which are related to coagulation status. Through dimensionality reduction of these results, we constructed the CHSS prognostic score to comprehensively reflect the value of the patients’ blood test results. As we predicted, CHSS demonstrated superior predictive ability and stability compared to NLR and PLR. CHSS is composed of CRP, lymphocytes, HB, albumin, glucose, HDL-C, LDL-C, and PLT, and it comprehensively reflects the patient’s inflammatory, immune, nutritional and metabolic status. The response of CHSS to the body’s inflammation and immune balance primarily comes from CRP, PLT and lymphocyte count. CRP is an acute-phase reactive protein synthesized by the liver when stimulated by inflammatory factors. Elevated CRP indicates the presence of a persistent inflammatory state, which can promote tumor growth and metastasis by enhancing angiogenesis, supporting tumor cell survival, and increasing invasiveness (41, 42).

Beyond inflammation, CHSS can also reflect the body’s lipid metabolism balance. The coefficients of LDL-C and HDL-C in CHSS are 0.498 and -0.605, respectively, with elevated LDL-C and reduced HDL-C associated with poor prognosis in STS patients. LDL-C is the lipoprotein primarily responsible for distributing cholesterol to extrahepatic tissues and body cells, while HDL-C is the smallest and densest lipoprotein in the blood, functioning to clear excess cholesterol through reverse transport (43, 44). Abnormally elevated LDL-C and reduced HDL-C may indicate an abnormal lipid metabolism, where tumor cells uptake and synthesize more cholesterol and fatty acids to support their growth and proliferation (45). Similarly, the glucose coefficient in CHSS is 0.708, suggesting that elevated blood glucose is associated with a worse prognosis in STS patients. This aligns with previous research showing that elevated blood glucose, even below the diagnostic threshold for diabetes, is related to poor outcomes in cancer patients (46). This may be due to the high-glucose environment upregulating the pathways related to the Warburg effect in tumor cells, promoting tumor growth and metastasis. Additionally, glucose can activate various signaling pathways involved in tumor cell proliferation, metastasis, and therapy resistance, promoting malignant phenotypes (47). Experimental results show that cancer cells exposed to supraphysiological glucose concentrations become more aggressive, further confirming this (46, 48).

Lastly, CHSS may also reflect the body’s nutritional status. The nutritional status of patients is also closely related to the prognosis of tumor patients (49). The coefficients for albumin and hemoglobin (HB) in CHSS are -0.513 and -0.782, respectively. Albumin is a chronic-phase protein commonly used to assess a patient’s nutritional status and the body’s protein synthesis capacity (15). An abnormal decrease in albumin indicates poor nutritional status and may weaken the body’s antitumor immune function and response to treatment (50). HB is the primary carrier responsible for transporting oxygen from the lungs to tissues throughout the body. Anemia may reflect a state of insufficient oxygen supply to tissues, and in a hypoxic environment, tumor tissues adapt by activating a series of survival-promoting mechanisms, such as inducing angiogenesis, which in turn promotes further tumor growth and metastasis (51, 52). Additionally, a decrease in HB may also indicate systemic malnutrition and cachexia in cancer patients.

In conclusion, we believe that CHSS utilizes the patient’s test results more comprehensively, reflecting the overall status of the body, and thus has a stronger predictive capability for patient prognosis. However, it must be acknowledged that our study has certain limitations. First, our study is a single-center retrospective study, which may introduce some selection bias. Secondly, although the CHSS exhibits stronger predictive capabilities, its computational complexity exceeds that of NLR and PLR, and the optimal coefficients for individual markers may vary across different cohorts, potentially limiting its clinical applicability. However, the enhanced stability of CHSS predictions provides a novel direction for developing next-generation biomarkers: integrating comprehensive indicators reflecting systemic inflammatory, immune, and nutritional statuses can circumvent the risk of prediction failure inherent to single-marker reliance. Future studies should focus on identifying the optimal balance between computational complexity and predictive performance in multicenter, large-scale cohorts. Furthermore, the development of standardized assays analogous to Oncotype DX is critical to advancing the clinical translation of CHSS (53). Lastly, our study only included the most common laboratory test results, and some novel test results with prognostic value, such as cytokines test, may have been overlooked.

## 5 Conclusion

The CHSS, composed of multiple test results, demonstrates superior predictive ability and stability for overall survival in STS patients compared to NLR/PLR. CHSS is an independent risk factor for OS in STS patients. The nomogram based on CHSS aids in the personalized management of patients.

## Data Availability

The raw data supporting the conclusions of this article will be made available by the authors, without undue reservation.

## References

[1] Hoefkens F Dehandschutter C Somville J Meijnders P Van Gestel D. Soft tissue sarcoma of the extremities: pending questions on surgery and radiotherapy. Radiat Oncol (London England). (2016) 11:136. doi: 10.1186/s13014-016-0668-9, PMID: 27733179 PMC5062836

[2] Siegel RL Miller KD Wagle NS Jemal A. Cancer statistics, 2023. CA: A Cancer J clinicians. (2023) 73:17–48. doi: 10.3322/caac.21763, PMID: 36633525

[3] Fornaciari G. Histology of ancient soft tissue tumors: A review. Int J paleopathology. (2018) 21:64–76. doi: 10.1016/j.ijpp.2017.02.007, PMID: 29776878

[4] Mastrangelo G Coindre JM Ducimetière F Dei Tos AP Fadda E Blay JY. Incidence of soft tissue sarcoma and beyond: a population-based prospective study in 3 European regions. Cancer. (2012) 118:5339–48. doi: 10.1002/cncr.27555, PMID: 22517534

[5] Lindberg RD Martin RG Romsdahl MM Barkley HT Jr. Conservative surgery and postoperative radiotherapy in 300 adults with soft-tissue sarcomas. Cancer. (1981) 47:2391–7. doi: 10.1002/1097-0142(19810515)47:10<2391::AID-CNCR2820471012>3.0.CO;2-B, PMID: 7272893

[6] Judson I Verweij J Gelderblom H Hartmann JT Schöffski P Blay JY. Doxorubicin alone versus intensified doxorubicin plus ifosfamide for first-line treatment of advanced or metastatic soft-tissue sarcoma: a randomised controlled phase 3 trial. Lancet Oncology. (2014) 15:415–23. doi: 10.1016/s1470-2045(14)70063-4, PMID: 24618336

[7] Italiano A Mathoulin-Pelissier S Cesne AL Terrier P Bonvalot S Collin F. Trends in survival for patients with metastatic soft-tissue sarcoma. Cancer. (2011) 117:1049–54. doi: 10.1002/cncr.25538, PMID: 20945333

[8] Gamboa AC Gronchi A Cardona K. Soft-tissue sarcoma in adults: An update on the current state of histiotype-specific management in an era of personalized medicine. CA: A Cancer J clinicians. (2020) 70:200–29. doi: 10.3322/caac.21605, PMID: 32275330

[9] Greten FR Grivennikov SI. Inflammation and cancer: triggers, mechanisms, and consequences. Immunity. (2019) 51:27–41. doi: 10.1016/j.immuni.2019.06.025, PMID: 31315034 PMC6831096

[10] Martínez-Garay C Djouder N. Dietary interventions and precision nutrition in cancer therapy. Trends Mol medicine. (2023) 29:489–511. doi: 10.1016/j.molmed.2023.04.004, PMID: 37263858

[11] DeBerardinis RJ Chandel NS. Fundamentals of cancer metabolism. Sci advances. (2016) 2:e1600200. doi: 10.1126/sciadv.1600200, PMID: 27386546 PMC4928883

[12] Cupp MA Cariolou M Tzoulaki I Aune D Evangelou E Berlanga-Taylor AJ. Neutrophil to lymphocyte ratio and cancer prognosis: an umbrella review of systematic reviews and meta-analyses of observational studies. BMC medicine. (2020) 18:360. doi: 10.1186/s12916-020-01817-1, PMID: 33213430 PMC7678319

[13] Diem S Schmid S Krapf M Flatz L Born D Jochum W. Neutrophil-to-Lymphocyte ratio (NLR) and Platelet-to-Lymphocyte ratio (PLR) as prognostic markers in patients with non-small cell lung cancer (NSCLC) treated with nivolumab. Lung Cancer (Amsterdam Netherlands). (2017) 111:176–81. doi: 10.1016/j.lungcan.2017.07.024, PMID: 28838390

[14] Shi J Liu T Ge Y Liu C Zhang Q Xie H. Cholesterol-modified prognostic nutritional index (CPNI) as an effective tool for assessing the nutrition status and predicting survival in patients with breast cancer. BMC medicine. (2023) 21:512. doi: 10.1186/s12916-023-03225-7, PMID: 38129842 PMC10740286

[15] Liang Y Hou T Que Y Zhao B Xiao W Zhang X. Elevated controlling nutritional status (CONUT) score is associated with poor long-term survival in patients with low-grade soft-tissue sarcomas treated with surgical resection. Clin orthopaedics related Res. (2019) 477:2287–95. doi: 10.1097/corr.0000000000000767, PMID: 31107315 PMC6999946

[16] Kusama H Kittaka N Soma A Taniguchi A Kanaoka H Nakajima S. Predictive factors for response to neoadjuvant chemotherapy: inflammatory and immune markers in triple-negative breast cancer. Breast Cancer (Tokyo Japan). (2023) 30:1085–93. doi: 10.1007/s12282-023-01504-y, PMID: 37782377

[17] Ogiwara T Kawazoe H Egami S Hashimoto H Saito Y Sakiyama N. Prognostic value of baseline medications plus neutrophil-to-lymphocyte ratio in the effectiveness of nivolumab and pembrolizumab in patients with advanced non-small-cell lung cancer: A retrospective study. Front oncology. (2021) 11:770268. doi: 10.3389/fonc.2021.770268, PMID: 34820333 PMC8606521

[18] Adamowicz K Romanowska A Zaucha RE. Prognostic value of the neutrophil-to-lymphocyte ratio (NLR) in advanced non-small cell lung cancer. Ann Oncol. (2018) 29:viii517. doi: 10.1093/annonc/mdy292.055

[19] Rimando J Campbell J Kim JH Tang SC Kim S. The pretreatment neutrophil/lymphocyte ratio is associated with all-cause mortality in black and white patients with non-metastatic breast cancer. Front oncology. (2016) 6:81. doi: 10.3389/fonc.2016.00081, PMID: 27064712 PMC4815293

[20] Liu C Li L Lu WS Du H Yan LN Yang JY. Neutrophil-lymphocyte ratio plus prognostic nutritional index predicts the outcomes of patients with unresectable hepatocellular carcinoma after transarterial chemoembolization. Sci reports. (2017) 7:13873. doi: 10.1038/s41598-017-13239-w, PMID: 29066730 PMC5654965

[21] Sengul D Sengul I. Are there any variation in neutrophil lymphocyte ratio, mean platelet volume, and platelet count between papillary thyroid cancer and benign nodular thyroid diseases? Sanamed. (2018) 13:11–6. doi: 10.24125/sanamed.v13i1.209

[22] Sengul D Sengul I. IS THERE ANY LINK BETWEEN A KIND OF THYROCYTE DYSFUNCTION, HYPOTHYROIDISM, AND INFLAMMATORY HEMATOLOGIC PARAMETERS IN THE CASES POSSESSING THE BENIGN THYROID NODULES? A 5-YEAR SINGLE-CENTRE EXPERIENCE. J SANAMED. (2018) 13. doi: 10.24125/sanamed.v13i1.211

[23] Li LQ Bai ZH Zhang LH Zhang Y Lu XC Zhang Y. Meta-analysis of hematological biomarkers as reliable indicators of soft tissue sarcoma prognosis. Front oncology. (2020) 10:30. doi: 10.3389/fonc.2020.00030, PMID: 32082998 PMC7002470

[24] Sengul I Sengul D. Deep soft tissue leiomyoma of the lower extremities: a case report. Acta chirurgica Belgica. (2009) 109:104–5. doi: 10.1080/00015458.2009.11680386, PMID: 27416298

[25] Sengul D Sengul I Ustun H. Dedifferentiated liposarcoma of the left thigh: a rare case. Med Arch (Sarajevo Bosnia Herzegovina). (2019) 73:121–2. doi: 10.5455/medarh.2019.73.121.122, PMID: 31391701 PMC6643339

[26] Viñal D Martinez D Garcia-Cuesta JA Gutierrez-Sainz L Martinez-Recio S Villamayor J. Prognostic value of neutrophil-to-lymphocyte ratio and other inflammatory markers in patients with high-risk soft tissue sarcomas. Clin Trans oncology: Off Publ Fed Spanish Oncol Societies Natl Cancer Institute Mexico. (2020) 22:1849–56. doi: 10.1007/s12094-020-02324-8, PMID: 32125644

[27] Idowu OK Ding Q Taktak AF Chandrasekar CR Yin Q. Clinical implication of pretreatment neutrophil to lymphocyte ratio in soft tissue sarcoma. Biomarkers: Biochem Indic exposure response susceptibility to chemicals. (2012) 17:539–44. doi: 10.3109/1354750x.2012.699554, PMID: 22793493

[28] Szkandera J Gerger A Liegl-Atzwanger B Absenger G Stotz M Friesenbichler J. The lymphocyte/monocyte ratio predicts poor clinical outcome and improves the predictive accuracy in patients with soft tissue sarcomas. Int J cancer. (2014) 135:362–70. doi: 10.1002/ijc.28677, PMID: 24347236

[29] Fausti V De Vita A Vanni S Ghini V Gurrieri L Riva N. Systemic inflammatory indices in second-line soft tissue sarcoma patients: focus on lymphocyte/monocyte ratio and trabectedin. Cancers. (2023) 15. doi: 10.3390/cancers15041080, PMID: 36831421 PMC9954182

[30] Wang Y Peng C Cheng Z Wang X Wu L Li J. The prognostic significance of preoperative neutrophil-lymphocyte ratio in patients with hepatocellular carcinoma receiving hepatectomy: A systematic review and meta-analysis. Int J Surg (London England). (2018) 55:73–80. doi: 10.1016/j.ijsu.2018.05.022, PMID: 29787804

[31] Senft D. Inflammatory neutrophils. Nat Rev Cancer. (2023) 23:801. doi: 10.1038/s41568-023-00644-9, PMID: 37946086

[32] Chen H Zhou XH Li JR Zheng TH Yao FB Gao B. Neutrophils: Driving inflammation during the development of hepatocellular carcinoma. Cancer letters. (2021) 522:22–31. doi: 10.1016/j.canlet.2021.09.011, PMID: 34517084

[33] Paijens ST Vledder A de Bruyn M Nijman HW. Tumor-infiltrating lymphocytes in the immunotherapy era. Cell Mol Immunol. (2021) 18:842–59. doi: 10.1038/s41423-020-00565-9, PMID: 33139907 PMC8115290

[34] Koukourakis MI Giatromanolaki A. Lymphopenia and intratumoral lymphocytic balance in the era of cancer immuno-radiotherapy. Crit Rev oncology/hematology. (2021) 159:103226. doi: 10.1016/j.critrevonc.2021.103226, PMID: 33482348

[35] Chan JY Zhang Z Chew W Tan GF Lim CL Zhou L. Biological significance and prognostic relevance of peripheral blood neutrophil-to-lymphocyte ratio in soft tissue sarcoma. Sci Rep. (2018) 8:11959. doi: 10.1038/s41598-018-30442-5, PMID: 30097600 PMC6086886

[36] Que H Fu Q Lan T Tian X Wei X. Tumor-associated neutrophils and neutrophil-targeted cancer therapies. Biochim Biophys Acta Rev cancer. (2022) 1877:188762. doi: 10.1016/j.bbcan.2022.188762, PMID: 35853517

[37] Zhao Y Rahmy S Liu Z Zhang C Lu X. Rational targeting of immunosuppressive neutrophils in cancer. Pharmacol Ther. (2020) 212:107556. doi: 10.1016/j.pharmthera.2020.107556, PMID: 32343986

[38] Qi X Chen J Wei S Ni J Song L Jin C. Prognostic significance of platelet-to-lymphocyte ratio (PLR) in patients with breast cancer treated with neoadjuvant chemotherapy: a meta-analysis. BMJ Open. (2023) 13:e074874. doi: 10.1136/bmjopen-2023-074874, PMID: 37996220 PMC10668253

[39] Goubran HA Burnouf T Radosevic M El-Ekiaby M. The platelet-cancer loop. Eur J Internal medicine. (2013) 24:393–400. doi: 10.1016/j.ejim.2013.01.017, PMID: 23433737

[40] Gay LJ Felding-Habermann B. Contribution of platelets to tumour metastasis. Nat Rev Cancer. (2011) 11:123–34. doi: 10.1038/nrc3004, PMID: 21258396 PMC6894505

[41] Lin ZY Liang ZX Zhuang PL Chen JW Cao Y Yan LX. Intrahepatic cholangiocarcinoma prognostic determination using pre-operative serum C-reactive protein levels. BMC cancer. (2016) 16:792. doi: 10.1186/s12885-016-2827-7, PMID: 27733196 PMC5059936

[42] Minichsdorfer C Gleiss A Aretin MB Schmidinger M Fuereder T. Serum parameters as prognostic biomarkers in a real world cancer patient population treated with anti PD-1/PD-L1 therapy. Ann medicine. (2022) 54:1339–49. doi: 10.1080/07853890.2022.2070660, PMID: 35535695 PMC9103267

[43] Cui F Zhou H Lv D Wen J Gong Q Rong Y. Preoperative serum low-density lipoprotein cholesterol is an independent prognostic factor in patients with renal cell carcinoma after nephrectomy. Lipids Health disease. (2023) 22:26. doi: 10.1186/s12944-023-01791-6, PMID: 36814277 PMC9945686

[44] Liao F He W Jiang C Yin C Guo G Chen X. A high LDL-C to HDL-C ratio predicts poor prognosis for initially metastatic colorectal cancer patients with elevations in LDL-C. OncoTargets Ther. (2015) 8:3135–42. doi: 10.2147/ott.S90479, PMID: 26604782 PMC4629979

[45] Xiao B Ouyang H Gulizeba H Fu H Wang Z Huang Y. Nomogram for predicting the prognosis of metastatic colorectal cancer patients treated with anti-PD1 therapy based on serum lipids analysis. Cancer immunology immunotherapy: CII. (2023) 72:3683–92. doi: 10.1007/s00262-023-03519-y, PMID: 37589756 PMC10576722

[46] Ramteke P Deb A Shepal V Bhat MK. Hyperglycemia associated metabolic and molecular alterations in cancer risk, progression, treatment, and mortality. Cancers. (2019) 11. doi: 10.3390/cancers11091402, PMID: 31546918 PMC6770430

[47] Supabphol S Seubwai W Wongkham S Saengboonmee C. High glucose: an emerging association between diabetes mellitus and cancer progression. J Mol Med (Berlin Germany). (2021) 99:1175–93. doi: 10.1007/s00109-021-02096-w, PMID: 34036430

[48] Ryu TY Park J Scherer PE. Hyperglycemia as a risk factor for cancer progression. Diabetes Metab J. (2014) 38:330–6. doi: 10.4093/dmj.2014.38.5.330, PMID: 25349819 PMC4209346

[49] Sasaki H Nagano S Komiya S Taniguchi N Setoguchi T. Validation of different nutritional assessment tools in predicting prognosis of patients with soft tissue spindle-cell sarcomas. Nutrients. (2018) 10. doi: 10.3390/nu10060765, PMID: 29899304 PMC6024570

[50] Olpe T Wunderle C Bargetzi L Tribolet P Laviano A Stanga Z. Muscle matters: Prognostic implications of malnutrition and muscle health parameters in patients with cancer. A secondary analysis of a randomised trial. Clin Nutr (Edinburgh Scotland). (2024) 43:2255–62. doi: 10.1016/j.clnu.2024.07.020, PMID: 39181036

[51] Caro JJ Salas M Ward A Goss G. Anemia as an independent prognostic factor for survival in patients with cancer: a systemic, quantitative review. Cancer. (2001) 91:2214–21. doi: 10.1002/1097-0142(20010615)91:12<2214::AID-CNCR1251>3.0.CO;2-P, PMID: 11413508

[52] Chen X Zhou H Lv J. The importance of hypoxia-related to hemoglobin concentration in breast cancer. Cell Biochem biophysics. (2024) 82:1893–906. doi: 10.1007/s12013-024-01386-7, PMID: 38955926

[53] Berton Giachetti PPM Carnevale Schianca A Trapani D Marra A Toss A Marchiò C. Current controversies in the use of Oncotype DX in early breast cancer. Cancer Treat Rev. (2025) 135. doi: 10.1016/j.ctrv.2025.102887, PMID: 40048856

